# Simultaneous co-infection with swine influenza A and porcine reproductive and respiratory syndrome viruses potentiates adaptive immune responses

**DOI:** 10.3389/fimmu.2023.1192604

**Published:** 2023-05-23

**Authors:** Tiphany Chrun, Emmanuel A. Maze, Kelly J. Roper, Eleni Vatzia, Basudev Paudyal, Adam McNee, Veronica Martini, Tanuja Manjegowda, Graham Freimanis, Adrian Silesian, Noemi Polo, Becky Clark, Emily Besell, Georges Booth, Brigid Veronica Carr, Matthew Edmans, Alejandro Nunez, Surapong Koonpaew, Nanchaya Wanasen, Simon P. Graham, Elma Tchilian

**Affiliations:** ^1^The Pirbright Institute, Woking, United Kingdom; ^2^Pathology and Animal Sciences, Animal and Plant Health Agency, Addlestone, United Kingdom; ^3^Virology and Cell Technology Laboratory, National Center for Genetic Engineering and Biotechnology (BIOTEC), National Science and Technology Development Agency, Pathumthani, Thailand

**Keywords:** viral co-infection, swine influenza A virus, porcine reproductive and respiratory syndrome virus, pig, immune response

## Abstract

Porcine respiratory disease is multifactorial and most commonly involves pathogen co-infections. Major contributors include swine influenza A (swIAV) and porcine reproductive and respiratory syndrome (PRRSV) viruses. Experimental co-infection studies with these two viruses have shown that clinical outcomes can be exacerbated, but how innate and adaptive immune responses contribute to pathogenesis and pathogen control has not been thoroughly evaluated. We investigated immune responses following experimental simultaneous co-infection of pigs with swIAV H3N2 and PRRSV-2. Our results indicated that clinical disease was not significantly exacerbated, and swIAV H3N2 viral load was reduced in the lung of the co-infected animals. PRRSV-2/swIAV H3N2 co-infection did not impair the development of virus-specific adaptive immune responses. swIAV H3N2-specific IgG serum titers and PRRSV-2-specific CD8β^+^ T-cell responses in blood were enhanced. Higher proportions of polyfunctional CD8β^+^ T-cell subset in both blood and lung washes were found in PRRSV-2/swIAV H3N2 co-infected animals compared to the single-infected groups. Our findings provide evidence that systemic and local host immune responses are not negatively affected by simultaneous swIAV H3N2/PRRSV-2 co-infection, raising questions as to the mechanisms involved in disease modulation.

## 1 Introduction

Porcine respiratory disease is a major cause of economic losses in the global pig industry, hampering growth and production (1). The etiology of this disease is often multifactorial, leading to its description as the porcine respiratory disease complex (PRDC) (2). The pathogens involved in the PRDC include both bacteria and viruses, and co-infections are identified in over 80% of cases (3). Major bacterial contributors are *Actinobacillus pleuropneumoniae*, *Mycoplasma hyopneumoniae*, *Bordetella bronchiseptica*, *Pasteurella multocida*, *Hemophilus parasuis*, *Streptococcus suis*, and *Glaesserella parasuis*; viruses include porcine reproductive and respiratory syndrome virus (PRRSV), swine influenza A virus (swIAV), porcine circovirus type 2 (PCV2), and porcine respiratory alphacoronavirus (PRCV) (3, 4). However, PRRSV and swIAV are most commonly detected in PRDC-affected piglets (3).

IAV is a single-stranded, negative-sense RNA virus harboring eight genome segments, classified in the *Articulavirales* order, *Orthomyxoviridae* family, and *Alphainfluenzavirus* genus (5). With the ability to infect a range of species including birds, horses, pigs, and humans, IAV is a major threat to animal and human health (6). The pig is susceptible to both avian and human IAVs and can serve as a “mixing vessel” capable of generating reassortant strains with the potential to cause significant outbreaks (7). Epizootic swine influenza is mainly caused by swIAV subtypes H1N1, H1N2, and H3N2, which primarily infect epithelial cells of the upper and lower respiratory tract (6). Clinical symptoms may be inapparent or include cough, fever, and nasal discharge, depending on strain virulence, host factors, age, and genetic background (8). PRRSV is a single-stranded, positive-sense RNA virus belonging to the *Nidovirales* order, the *Arteriviridae* family, and the *Variarterivirinae* subfamily (5). PRRSV exists as two species, PRRSV-1 (*Betaarterivirus suid 1*), which predominates in Europe, and PRRSV-2 (*B. suid 2*), which predominates in the Americas and Asia (9). PRRSV is a myelotropic virus with a host range restricted to *Sus scrofa*, and as of today, PRRSV remains an important pathogen circulating in pig herds globally, causing late-term gestational failures in sows and respiratory disorders, growth reduction, and mortality in piglets (10). Like swine influenza, symptoms may vary based on strain virulence and host factors (10).

While it is difficult to cover all possible scenarios in co-infection experiments, studies have been conducted to examine the consequences of swIAV and PRRSV co-infections on pathology and vaccine immunity using simultaneous or sequential infection protocols. SwIAV and PRRSV co-infections can be subclinical (11–13) or detrimental (14–16) and may compromise clinical protection conferred by PRRSV-2 and swIAV vaccines (12, 14). The consequences of co-infection on innate and adaptive immune responses appear similarly complex. Concurrent co-infection with swIAV and PRRSV increased the secretion of IL-6 and IL-10 by bronchoalveolar lavage cells (17) and IL-12 gene expression in lung tissue (12) but did not affect the kinetics or magnitude of antigen-specific proliferating immune cells (supposedly T cells) or antibody responses to swIAV and PRRSV (13, 14). During swIAV and PRRSV superinfection (infection with swIAV 1 week after PRRSV), higher anti-swIAV antibody responses were detected in the lungs, and PRRSV-specific cell-mediated immune responses were detected earlier in the blood (11). However, an in-depth analysis of the T-cell responses to both infecting agents has not been assessed so far.

In this report, we investigated the clinical, virological, and immunological outcomes following simultaneous swIAV H3N2 and PRRSV-2 co-infection. We analyzed the whole blood transcriptomic signature at 5 days post-infection, the magnitude of antibody responses, and the quality of T-cell responses against both viruses up to 41 days after infection.

## 2 Materials and methods

### 2.1 Propagation and titration of viruses

SwIAV H3N2 CM5 isolate (A/swine/Thailand/CM5/2018; referred to herein as H3N2) and PRRSV-2 (16CB02; (referred to herein as PRRSV-2) were propagated and titrated in Madin–Darby canine kidney (MDCK) and in African green monkey kidney cells (MARC-145), respectively, as previously described (12). MDCK cells were cultured in Eagle’s minimum essential medium (MEM; Merck, Felltham, UK) supplemented with 10% heat-inactivated fetal bovine serum (HI FBS; Thermo Fisher Scientific, Loughborough, UK) and antibiotics (100 U/ml of penicillin and 100 µg/ml of streptomycin; Thermo Fisher) at 37°C in 5% CO_2_. MARC-145 cells were cultured in Dulbecco’s modified MEM (DMEM; Merck, Poole, UK) supplemented with 10% HI FBS and antibiotics at 37°C in 5% CO_2_.

### 2.2 Experimental infection of pigs

**Figure 1:**
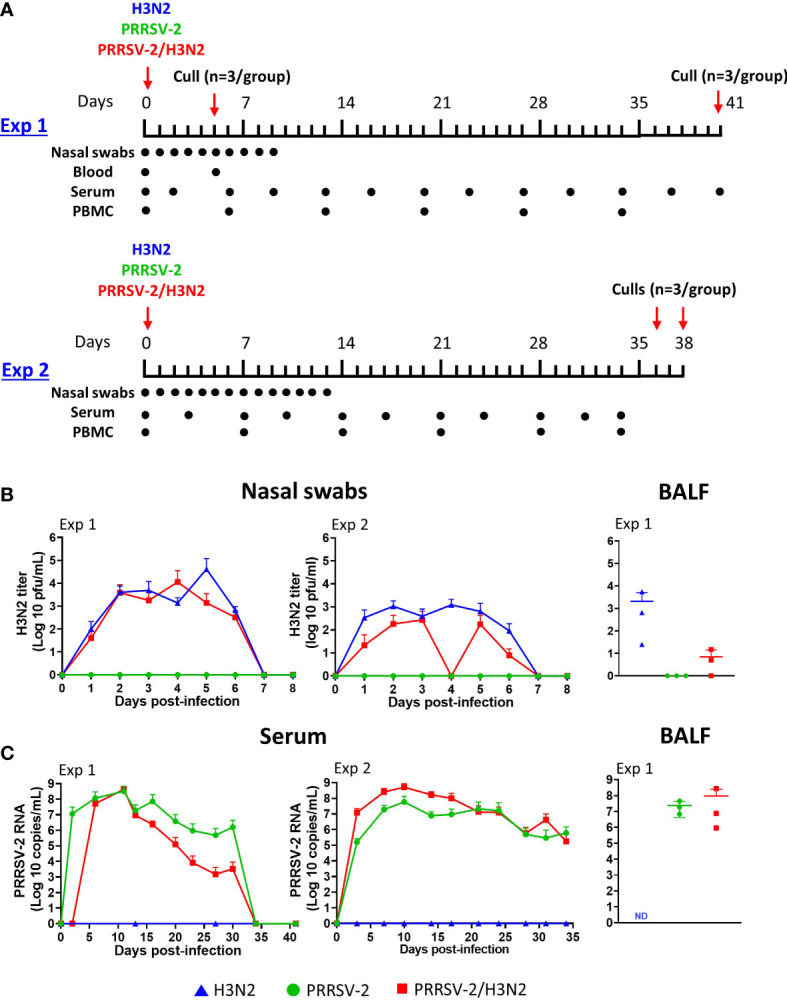
Effect of PRRSV-2 single infection and H3N2 co-infection on viral loads. **(A)** In the first experiment (Exp 1), eighteen 5–7-week-old pigs were randomly assigned to three groups (n = 6) and intranasally challenged with 1 × 10^6^ pfu of H3N2, 10^5^ TCID_50_ PRRSV-2, or simultaneously with 1 × 10^6^ pfu of H3N2 and 10^5^ TCID_50_ PRRSV-2. In a second experiment (Exp 2), a higher dose of H3N2 at 5 × 10^6^ pfu was used in the single-infected and PRRSV-2/H3N2 co-infected animals. Pigs were culled to assess lung lesions at 5 dpi (n = 3 in Exp 1) or kept until ~6 weeks post-challenge (n = 3 in Exp 1 and n = 6 in Exp 2). Nasal swabs and blood samples were collected on the indicated day by a dot at the corresponding timepoints for both experiments. **(B)** H3N2 titers (pfu/ml) in nasal swabs and bronchoalveolar lavage fluid (BALF) were measured by plaque assay. **(C)** PRRSV-2 viral RNA copy numbers/ml in serum and BALF were assessed by qRT-PCR specific to PRRSV-2 ORF7. The mean values of viral loads in nasal swabs and sera for each group ± SD are indicated (n = 3 per group from 5 dpi in Exp 1 or n = 6 per group in Exp 2). Individual values of viral loads in BALF are represented by a symbol, and the mean is indicated by a horizontal bar (n = 3 per group). Comparisons between two groups were performed using the Mann–Whitney test. ND, not done.

Animal experiments were approved by the Animal Welfare and Ethical Review Board of The Pirbright Institute. All animals were housed in a high biocontainment animal facility at The Pirbright Institute, and all procedures were performed in accordance with the UK Animal (Scientific Procedures) Act 1986 supported by the project license P6F09D691. Two animal experiments were conducted (**Figure 1A**): a pilot study (Exp 1) was conducted to characterize the immunopathology of the field-isolated viral strains in pigs and the innate and adaptive immune responses following single infections and co-infections with H3N2 and PRRSV-2. A second study (Exp 2) was performed to confirm the observations and to further analyze the T-cell responses. In both studies, 5–7-week-old, Large White-Landrace-Hampshire crossbred, female pigs were sourced from a high-health-status commercial herd. Animals were tested negative for infection to IAV and PRRSV prior to their arrival as previously described (12). Pigs were randomly assigned to three groups of six pigs each and were acclimatized for at least 6 days.

In both experiments, the same batches of viral stocks were used. In Exp 1, pigs were inoculated intranasally with 1 × 10^6^ pfu of H3N2, 1 × 10^5^ TCID_50_ PRRSV-2, or simultaneously with 1 × 10^6^ pfu of H3N2 and 1 × 10^5^ TCID_50_ PRRSV-2 (PRRSV-2/H3N2) diluted in 4 ml of DMEM (Thermo Fisher Scientific) using a mucosal atomization device (2 ml/nostril, MAD 300, Wolfe Tory Medical, Salt Lake City, UT, USA). Half of the pigs (n = 3) in each group were euthanized at 5 days post-infection (dpi) to assess the gross and histopathological lung lesions. Clinical signs (**Supplementary Table 1**) and rectal temperatures were monitored daily until the end of the study at 41 dpi. The incidence of pyrexia (rectal temperatures >40°C) was calculated as a percentage of the total number of rectal temperatures measured within a group. Blood samples were collected for whole blood transcriptomic analysis at 0 and 5 dpi, for peripheral blood mononuclear cell (PBMC) isolation every week, and for serum isolation twice a week. Nasal swabs were collected daily for 10 days. At day 41 dpi, pigs were humanely euthanized with an overdose of pentobarbital sodium anesthetic.

In Exp 2, a similar protocol of experimental challenge and sampling was used, except that pigs were inoculated intranasally with a dose of 5 × 10^6^ pfu of H3N2, 1 × 10^5^ TCID_50_ PRRSV-2, or simultaneously with 5 × 10^6^ pfu of H3N2 and 1 × 10^5^ TCID_50_ PRRSV-2 diluted in 4 ml of DMEM using the MAD 300. Compared to that in Exp 1, a higher dose of H3N2 was employed to confirm exacerbation of clinical signs following PRRSV-2/H3N2 co-infection. Clinical signs, rectal body temperatures, and samplings were performed as described in Exp 1. Nasal swabs were collected daily for 13 days. Animals were humanely euthanized with an overdose of pentobarbital sodium anesthetic at 36 and 38 dpi.

### 2.3 Gross pathology and histopathological examination of lungs

Macroscopic and histopathological analyses of the lungs were conducted as previously described (18–21). Digital photographs of the dorsal and ventral lungs were taken. Macroscopic lesions were scored blindly as per Halbur et al. (1995), and an additional quantitative scoring was performed by determining the percentage of the lung displaying gross lesions using digital photographs and ImageJ image analysis software. A small piece of the cranial, middle, and caudal lobes was removed and immersed into 10% neutral buffered formalin for histological processing. Formalin-fixed tissues were paraffin wax embedded, and 4-μm sections were cut for hematoxylin and eosin staining or immunohistochemical straining of IAV or PRRSV nucleoprotein. Lesions were scored blind using the “Morgan score” (20), which evaluates the severity of necrosis of the bronchiolar epithelium, airway inflammation, perivascular/bronchiolar cuffing, alveolar exudates, and septal inflammation. The “Iowa” scoring system, which incorporates the presence of viral antigen in the scoring, was also included (18).

### 2.4 Sample processing and cell isolation

For transcriptome analysis, blood was collected at 0 and 5 dpi into PAXgene blood RNA tubes (PreAnalytiX, Qiagen, Manchester, UK) according to the manufacturer’s instruction and frozen at −80°C until further use. Serum, nasal swabs, PBMCs, and bronchoalveolar lavage fluid (BALF) and cells (BALCs) were collected and processed as previously described (12, 22). Freshly isolated cells were used for IFN-γ ELISpot assay (Exp 1) or were cryopreserved in 10% dimethyl sulfoxide (DMSO) with 90% HI FBS for immunoassays (Exp 2).

### 2.5 RNA extraction from fluids

Total RNA extraction from blood samples collected in PAXgene tubes was performed using the PAXgene Blood RNA Kit (Qiagen) according to the manufacturer’s protocol. RNA was extracted from BALF and serum using the QIAamp Viral RNA kit (Qiagen) according to the manufacturer’s instructions. All extracted RNA samples were stored at −80°C until use.

### 2.6 SwIAV H3N2 titration and PRRSV detection by quantitative RT-PCR

SwIAV titers in nasal swabs and BALF were determined by plaque assay and expressed as pfu/ml as previously reported (12). PRRSV RNA was measured by reverse transcription quantitative PCR using the one-step QuantiTect Probe RT-PCR Kit (Qiagen) for samples collected from Exp 1 or QuantiNova Probe RT-PCR kit (Qiagen) for samples collected in Exp 2. A PRRSV-2 ORF7 RNA standard, PRRSV-2 ORF7-specific primers, and TaqMan probe were designed as previously described (12). The qRT-PCR was performed with 5 µl of the eluted samples and 45 µl of the master mix for the one-step QuantiTect Probe RT-PCR Kit or 5 µl of the master mix for the QuantiNova Probe RT-PCR kit. Samples and standards were run in duplicate under cycling conditions indicated by the supplier on a Stratagene Mx3500P cycler (Agilent Technologies, Santa Clara, CA, USA). The viral genome copy numbers were determined by interpolation of the standard curve.

### 2.7 RNA-sequencing and data analysis

Prior to sequencing library preparation, RNA quality was analyzed using an RNA ScreenTape on a TapeStation 4200 (Agilent Technologies, Santa Clara, CA, USA), and samples were quantified using the Qubit RNA BR assay kit (Thermo Fisher Scientific). Samples had good RNA integrity number (RIN) values ranging from 6.7 to 8.3, apart from one 5 dpi sample from the co-infection group (pig 74), which was excluded from the analysis due to poor RNA quality. Library preparation was performed in duplicate with an input of 1 µg of total RNA using the Illumina TruSeq Stranded mRNA Prep kit (Illumina, San Diego, CA, USA) according to the manufacturer’s instructions and automated using a Hamilton NGS Star (Hamilton, Bonaduz, Switzerland). Final library quality control was determined using the D1000 ScreenTape on a TapeStation 4200 (Agilent Technologies) for size distribution and the Qubit dsDNA BR kit (Thermo Fisher Scientific) for quantification. Libraries were then normalized to 5 nM before being randomly split into two pools of eight and nine samples. The normalized pooled libraries were quantified using the Qubit dsDNA HS kit (Thermo Fisher Scientific) and the NEBNext Illumina Library Quant Kit for Illumina (NEB, Ipswich, MA, USA) and adjusted to 5 nM prior to final denaturation and dilution. Pooled libraries were sequenced on two 1 × 150 single-end sequencing runs on a NextSeq 550 System (Illumina) with a 1% PhiX (Illumina) spike-in.

Sequencing data were checked against sequencing artifacts, poor quality, and other anomalies using FastQC (23). Cutadapt (24) was used to clean the data. After that, Subread (25) was used to map the reads to the host genome reference (Ensembl Sscrofa11.1). Mapping quality was assessed by Qualimap (26, 27). FeatureCounts (28) were used to annotate (Ensembl Sscrofa11.1.104) these alignments. Unique exons were taken into account for further analysis performed using R with edgeR (29–31). To convert observed library sizes into effective library sizes, scaling factors were computed using the trimmed mean of M-values (32). Next, gene-wise dispersion (33) was assessed using the biological coefficient of variation (BCV) against gene abundance (31). Poisson Distance (34), as well as multidimensional scaling (35), was used to measure dissimilarity between counts. Statistical testing for differential gene expression (DGE) was performed by fitting a quasi-likelihood negative binomial generalized log-linear model to count data. Gene-wise F-tests for a given contrast were performed (29). Finally, filtered results with false discovery rate (FDR) (36) <0.05 and |logFC| > 1 were taken into consideration. Fastq files for all samples were submitted to the National Center for Biotechnology Information (NCBI) Sequence Read Archive database under reference PRJNA940926.

### 2.8 IFN-γ ELISpot assay

IFN-γ ELISpot assays were performed as previously described (21) with minor modifications. Briefly, MultiScreen-HA 96-well plates (Merck) coated with anti-porcine IFN-γ monoclonal antibody (mAb; clone P2G10; BD Biosciences, San Jose, CA, USA) were blocked in Roswell Park Memorial Institute (RPMI) medium (Thermo Fisher Scientific) supplemented with 10% HI FBS and antibiotics (complete RPMI) for 2 h at 37°C in 5% CO_2_. Freshly isolated PBMCs and BALCs (Exp 1) or thawed cryopreserved PBMCs (Exp 2) were seeded at a density of 5 × 10^5^ cells/wells in triplicate. Cells were restimulated with H3N2 or PRRSV-2 at a multiplicity of infection (MOI) of 3.1 and 3.3, respectively, in complete RPMI for 18 h at 37°C in 5% CO_2_. Cells cultured in complete RPMI or with 10 µg/ml of concanavalin A (ConA, Merck) were used as controls. IFN-γ-secreting cells were revealed and enumerated as described, using the biotinylated anti-porcine IFN-g detection mAb (clone P2C11, BD Biosciences) followed by the secondary streptavidin–alkaline phosphatase and BCIP/NBT reagent (21). Plates were automatically counted using ImmunoSpot Reader (Cellular Technology Limited, Ohio, USA). Results were expressed as the number of IFN-γ-producing cells per 10^6^ cells minus the average number of IFN-γ-producing cells per 10^6^ cells in medium-only stimulated wells.

### 2.9 Intracellular cytokine staining

Intracellular cytokine staining was performed as previously described (12). In brief, cryopreserved PBMCs and BALCs were thawed, rested for at least 3 h at 37°C in 5% CO_2_, and seeded at a density of 2 × 10^6^ cells/wells. Cells were stimulated with H3N2 or PRRSV-2 at an MOI of 0.1 in complete RPMI for 16 h at 37°C in 5% CO_2_. Cells cultured in complete RPMI served as negative controls. Cytokine secretion was blocked by adding BD GolgiPlug at 1:1,000 (BD Biosciences) into the wells for a further 4 h at 37°C in 5% CO_2_ before staining. Cells were stained using the following mAbs: CD3-PE mAb (clone BB23-8E6-8C, BD Biosciences), CD4-PerCP-Cy5.5 mAb (clone 74-12-4, BD Biosciences), CD8β-FITC mAb (clone PPT23, BD Biosciences), and LIVE/DEAD™ Fixable Near-IR Dead Cell Stain (Thermo Fisher Scientific). Cells were treated with the BD Cytofix/Cytoperm™ Fixation/Permeabilization Kit (BD Biosciences) before intracellular staining using the following mAbs: IFN-γ-Alexa Fluor 647 mAb (clone P2G10, BD Biosciences), TNF-α-Brilliant Violet 421 mAb (clone Mab11, BioLegend, San Diego, CA, USA), and anti-porcine IL-2 mAb (clone A150D3F1, Thermo Fisher Scientific) and the secondary anti-mouse IgG2a-PE-Cy7 (clone m2a-15F8, Thermo Fisher Scientific). Cells were analyzed using a MACSQuant Analyzer 10 Flow Cytometer (Miltenyi Biotec, Bisley, UK), and data were analyzed using FlowJo software version 10.6.2 (BD Biosciences). Compensation was set according to single-color staining controls. Fluorescence minus one (FMO) controls were used to set the gates. Cytokine production within CD4^+^ and CD8β^+^ T-cell subsets was calculated as the percentage of cytokine-expressing cells minus the background response detected in cells cultured in media only for each pig. Boolean gating was performed to determine the frequencies of each combination of cytokine-producing CD4^+^ and CD8β^+^ T cells. Virus-specific T-cell response kinetics in PBMCs were determined as the percentage of each cytokine-producing cell subset corrected with its corresponding frequency recorded at 0 dpi per animal.

### 2.10 ELISA

H3N2-specific and PRRSV-specific IgG titers were measured by ELISA as reported previously (12). Briefly, 96-well plates (Nunc MAXIsorp, Thermo Fisher Scientific) coated overnight at 4°C with an optimized concentration of H3N2 or lysate from MARC-145 infected with PRRSV-2 were blocked with phosphate-buffered saline (PBS) with 4% milk for 1 h at room temperature (RT). Serial twofold dilutions of HI serum or BALF were added in duplicates, starting from 1:40 or 1:2, respectively, in a blocking buffer for 1 h at RT. H3N2- or PRRSV-2-specific IgG was detected using goat-anti-pig IgG conjugated with horseradish peroxidase (HRP; Bio-Rad, Watford, UK) at 1:10,000 followed by 3,3′,5,5′-tetramethylbenzidine (TMB) substrate (Thermo Fisher Scientific) incubation for 5 min. The plates were read at 450 nm with the Cytation3 Imaging Reader (BioTek Instruments, Vermont, USA). Antibody endpoint titers were determined as the highest dilution giving twice the optical density (OD) of the negative control wells (H3N2- or PRRSV-2-infected cell lysate-coated wells).

### 2.11 Virus neutralization tests

To determine H3N2-specific neutralizing antibody titers, a microneutralization (MN) assay was performed as previously described with minor modifications (37). In brief, HI serum samples were serially diluted twofold in DMEM with a starting dilution at 1:20 in 96-well plates. Equal volumes of diluted serum were incubated with 2 × 10^4^ pfu of swIAV H3N2 CM5 in serum-free DMEM for 2 h at 37°C in 5% CO_2_. MDCK-SIAT-1 cells (expressing human 2,6-sialyltransferase) at 3 × 10^4^ cells/well were added to the serum/virus mix, and plates were incubated for 18 h at 37°C in 5% CO_2_. Each serum was tested in duplicate. Cells were fixed and permeabilized with 2% paraformaldehyde (PFA) 0.5% Triton X-100 in PBS for 30 min at 4°C. After being blocked with 1% bovine serum albumin in PBS, cells were stained with an anti-IAV nucleoprotein mAb (clone AA5H, Bio-Rad) followed by secondary goat anti-mouse IgG HRP (Thermo Fisher Scientific). TMB substrate was added into each well, and the reaction was stopped after 5 min by adding 1 M of sulfuric acid. Absorbance was measured at 450 nm on the Cytation3 Imaging Reader (BioTek Instruments, Agilent). Antibody endpoint titers were determined as the last reciprocal serum dilution that caused a 50% reduction of the mean OD value obtained with H3N2-infected MDCK-SIAT-1 cells without serum.

PRRSV neutralizing antibody titers were determined as previously described (12). In brief, serially twofold diluted HI serum was incubated with 400 TCID_50_ of PRRSV-2 16CB02 strain for 1 h at 37°C, and the serum/virus mix was then added to MARC-145 cell monolayers. After 3 days of incubation at 37°C, fixed and permeabilized cells were labeled with an anti-PRRSV nucleoprotein mAb (SDOW17-A, Rural Technologies, Brookings, SD, USA) followed by a secondary goat anti-mouse IgG conjugated to HRP (Thermo Fisher Scientific). PRRSV-2-infected cells were visualized using 3,3′-diaminobenzidine substrates (DAB; Vector Laboratories, Burlingame, CA, USA). Neutralizing antibody titers were calculated as the reciprocal serum dilution that neutralized viral infection in 100% of the wells.

### 2.12 Statistical analysis

Data were analyzed with GraphPad Prism 9.4.1 software. All data were subject to a normality test (Anderson–Darling test). As distribution was not normal, the unpaired non-parametric Kruskal–Wallis test was used to compare data between three groups (clinical scores and body temperatures). The Mann–Whitney test was used to compare data between two groups (virological and immunological data). To better summarize the effects of the co-infection on virus loads and immune responses, the area under the curve (AUC) values of virus shedding and H3N2- or PRRSV-2-specific immune response over the time course of infection were calculated (**Table 1**).

**Table 1:** Calculated area under the curve of virological and immunological parameters over the time course.

Parameters	Exp #	H3N2	PRRSV-2	PRRSV-2/H3N2
**H3N2 pfu/ml AUC (nasal shedding)**	1	5.10E+04	0	1.80E+04
	2	3.06E+03	0	8.87E+02*^a^
**PRRSV-2 RNA copy/ml AUC (viremia)**	1	0	6.00E+08	4.90E+08
	2	0	4.60E+08	3.70E+09**
**H3N2-specific IgG titer AUC (serum)**	1	58,316	0	175,823
	2	47,330	0	146,324*
**PRRSV-2-specific IgG titer AUC (serum)**	1	310	134,200	142,219
	2	255	15,427	14,899
**Neutralizing H3N2-specific IgG titer AUC (serum)**	1	15,147	1,203	45,856
	2	1,773	47	3,733*
**Neutralizing PRRSV-2-specific IgG titer AUC (serum)**	1	ND	ND	ND
	2	0	19	2
**IFN-γ-producing cells H3N2-specific response AUC (PBMC)**	1	3,040	1,831	5,436
	2	816	433	1,443
**H3N2-specific cytokine-producing cells (PBMC)**	2 − CD4^+^	4.3	0.8	4.7
	2 − CD8β^+^	5.9	3.3	9.6
**IFN-γ-producing cells PRRSV-2-specific response AUC (PBMC)**	1	171	13,400	12,207
	2	531	3,419	3,240
**PRRSV-2-specific cytokine-producing cells (PBMC)**	2 − CD4^+^	1.4	5.7	6.0
	2 − CD8β^+^	3.3	9.6	18.8*

Comparisons between two groups were made using the Mann–Whitney test. Asterisks indicate significant differences (*p < 0.05).

ND, not done; PBMC, peripheral blood mononuclear cell; AUC, area under the curve.

a H3N2 titers at 4 dpi in all groups were excluded from the analysis.

## 3 Results

### 3.1 Clinical signs, lung pathology, and viral loads

The clinical signs observed were overall mild after infection with swIAV H3N2 and mild to moderate with PRRSV-2 or following co-infection. None of the pigs reached the humane endpoints in either study. Few cases of pyrexia (rectal temperature >40°C) were recorded in each group during Exp 1 (1.3% in the H3N2 group, 3.3% in the PRRSV-2 group, and 2.6% in the co-infected group; **Supplementary Figure 1A**). A higher occurrence of fever was recorded in each group in Exp 2 (9.1% in the H3N2 group, 12% in the PRRSV-2 group, and 7.9% in the co-infected group; **Supplementary Figure 1A**). Overall, swIAV H3N2 infection was mostly asymptomatic. PRRSV-2 infection induced skin discoloration on ears and other body extremities, and lethargy in some animals. After co-infection, skin discoloration on ears and body, lethargy, inappetence, and slightly labored breathing were observed in some individuals. Overall, across both studies, the PRRSV-2/H3N2 co-infected groups had similar clinical scores as the single-infected groups (mean of clinical scores in the H3N2-, PRRSV-2-, and co-infected groups, Exp 1: 0.95 vs. 1.58 vs. 1.74; Exp 2: 1.85 vs. 1.96 vs. 2.00; **Supplementary Figure 1B**).

The lungs collected at 5 dpi in Exp 1 were examined for gross and microscopic lesions. Both single H3N2 and PRRSV-2 infections induced minimal macroscopic lung lesions (mean Halbur scores of 1.3 and 0.3, respectively), and the co-infection slightly increased the severity of lung lesions (mean of 2.6, **Supplementary Figure 2A**). Similarly, the proportion of affected areas was low in the single-infected groups (mean of 0.15% in the H3N2 group and 0.02% in the PRRSV-2 group) and was slightly increased in the co-infected group (mean of 0.28%, **Supplementary Figure 2A**). Histopathological analyses indicated that all animals, irrespective of the group displayed, mild-to-moderate bronchointerstitial pneumonia as previously reported with these virus strains (12). More severe lesions were primarily found in cranial and middle lobes in comparison to caudal lobes. Evaluation of the severity of microscopic lung lesions by two scoring methods indicated that there was a slight increase in lung lesions in the co-infected group (mean scores in the H3N2-, PRRSV-2-, and co-infected groups, Iowa scoring: 13.3 vs. 7.5 vs. 14.7; Morgan scoring: 14.7 vs. 10.7 vs. 16, **Supplementary Figure 3**).

Following single infections and co-infections with swIAV H3N2 and PRRSV-2, viral loads in nasal swabs, BALF, and serum were measured (**Figures 1B, C**). A similar pattern of H3N2 viral shedding was observed after a single infection and co-infection (**Figure 1B**). Virus titers reached a plateau at 2 dpi and remained steady up to 6 dpi before decreasing to undetectable levels by 8 dpi. The cumulative nasal swabs titers from 1 to 7 dpi (AUC) were not significantly affected by co-infection (**Table 1**). In Exp 2, the cumulative H3N2 shedding was assessed without considering measures taken on 4 dpi because of an unexpected absence of viral titer in the H3N2 group, most likely due to a technical issue during sampling. The resulting H3N2 AUC was still significantly lower in the co-infected group compared to the H3N2 single-infected group (*p* < 0.01, **Table 1**). A mean fourfold lower H3N2 titer was measured in the BALF (5 dpi) from co-infected pigs compared to the single-infected group (**Figure 1B**). Regarding PRRSV-2 RNA loads in sera, different kinetics were obtained (**Figure 1C**). In Exp 1, levels of PRRSV-2 RNA peaked at 11 dpi and decreased until 30 dpi, and no viral RNA was detected thereafter. In Exp 2, levels of PRRSV-2 RNA peaked at 10 dpi and decreased until the end of the study (34 dpi). Lower levels of PRRSV-2 RNA were measured in the serum from co-infected pigs across in Exp 1 (**Table 1**). In contrast, a significantly higher level of PRRSV-2 RNA was measured in Exp 2 in the co-infected group compared to the single-infected group (*p* < 0.01, **Table 1**). In BALF (5 dpi), the levels of PRRSV-2 RNA were comparable between the PRRSV-2-infected and co-infected groups (**Figure 1C**).

Collectively, these data indicate that PRRSV/H3N2 co-infection did not significantly exacerbate disease, and it appeared to have a beneficial effect by reducing H3N2 viral load in nasal swabs and BALF (at 5 dpi) compared to single H3N2-infected animals.

### 3.2 Whole blood RNA-seq analysis

To better understand host immune responses following PRRSV-2/H3N2 co-infection, transcriptomic analysis to determine differentially expressed genes was performed on whole blood collected at 0 and 5 dpi from single-infected (PRRSV-2, n = 3; H3N2, n = 3) or co-infected (n = 3 for 0 dpi and n = 2 for 5 dpi) groups from Exp 1. Significant inter-sample/animal variation was observed, and significantly differentially expressed genes were not observed between timepoints or infection groups (**Supplementary Figures 4**, **5**).

### 3.3 Antibody responses

**Figure 2:**
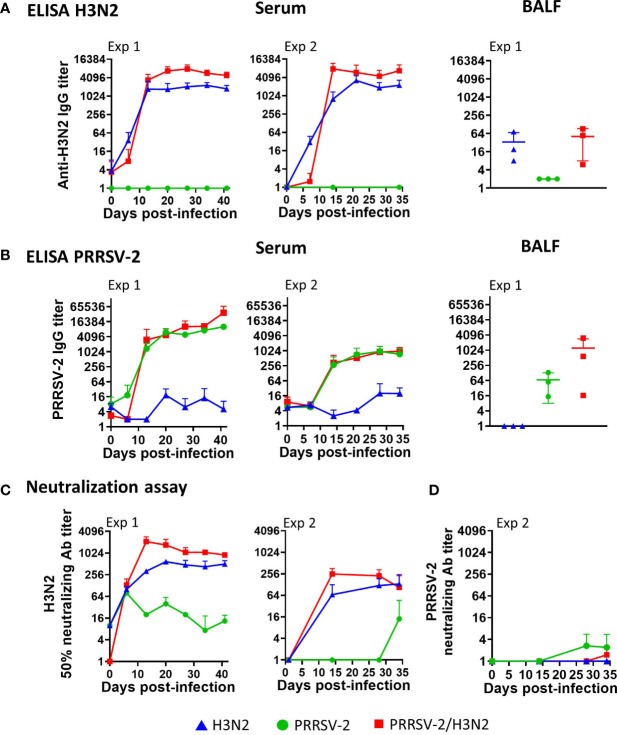
Effects of PRRSV-2/H3N2 co-infection on antibody responses. **(A)** H3N2-specific or **(B)** PRRSV-2 IgG titers in serum collected longitudinally (both Exp 1 and Exp 2) and in bronchoalveolar lavage fluid (BALF) collected at 41 dpi (Exp 1) were assessed by ELISA. **(C)** H3N2 and **(D)** PRRSV-2 neutralizing antibody titers in serum were assessed longitudinally by microneutralization (MN) and virus neutralization test (VNT). The mean value ± SD for each group (serum) or individual value and the mean in the indicated group (BALF) are represented. The H3N2 and PRRSV-2 loads’ area under the curve (**Table 1**) values were calculated over the time course for each animal. Comparisons between two groups were made using the Mann–Whitney test.

Both systemic (serum) and local (BALF) antibody responses to H3N2 and PRRSV-2 were assessed by ELISA and virus-neutralization assays (**Figure 2**). Following single infection and co-infection, H3N2-specific antibody titers increased in serum from 6 to 7 dpi and reached a plateau from 13 to 14 dpi, respectively, in Exp 1 and Exp 2 (**Figure 2A**). The H3N2 IgG titer was significantly higher in the co-infected group (*p* < 0.05, **Table 1**). Consistent with H3N2-binding antibody titers measured in serum, co-infected animals displayed higher H3N2 neutralizing antibody titers (**Figure 2C**; *p* < 0.05, **Table 1**). Low H3N2 neutralizing Ab titers were detected in serum from PRRSV-2-infected animals over the course of infection despite the absence of anti-H3N2 antibodies detected by ELISA in these sera (**Figure 2A**) and H3N2 viral load (**Figure 1B**). This observation likely reflects non-specific neutralization by the addition of a high serum concentration rather than cross-contamination during PRRSV-2 and H3N2 infections. In BALF at 5 dpi (Exp 1), the H3N2 IgG titers measured were similar in both single-infected and co-infected pigs (mean of 33 vs. 51, respectively).

With the use of PRRSV-2-MARC-145 cell lysate, low level reactivity was observed in serum from H3N2-infected pigs (**Figure 2B**). However, PRRSV-2-specific antibody titers increased from 6 to 7 dpi and reached a plateau at 14 dpi after the single infection and co-infection in both experiments (**Figure 2B**). Similar PRRSV-2-specific IgG titers were measured between the PRRSV-2-infected and co-infected groups (**Table 1**). PRRSV-2-specific IgG in BALF (Exp 1, 5 dpi) reached relatively high titers in 2/3 pigs following co-infection but not following the single infection (mean of IgG titers in the PRRSV-2-infected group vs. co-infected group, Exp 1: 68 vs. 1,229; **Figure 2B**). In Exp 2, PRRSV-2 neutralizing antibody titers in serum were detected earlier (28 dpi) and at a higher level in the single-infected animals compared to the co-infected animals (**Table 1**). PRRSV-2 neutralizing antibody titers were not measurable in Exp 1.

These results indicate that PRRSV-2/H3N2 co-infection enhanced serum H3N2-specific antibody responses, while PRRSV-2 antibody titers were unaffected.

### 3.4 H3N2-specific T-cell responses in peripheral blood

**Figure 3:**
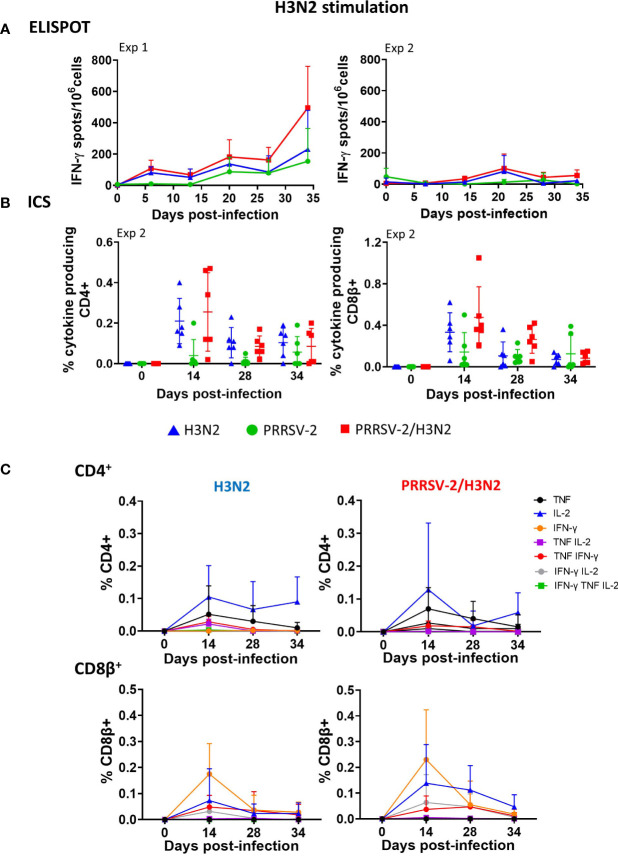
H3N2-specific polyfunctional T-cell responses in peripheral blood mononuclear cell (PBMC) following PRRSV-2/H3N2 co-infection. **(A)** IFN-γ secretion by PBMCs isolated weekly was determined by ELISpot assay. Cells were restimulated *in vitro* for 18 h with H3N2 (multiplicity of infection (MOI) 3.4) or cultured with medium. Corrected H3N2-specific IFN-γ-producing cells (minus unstimulated controls) were calculated per 10^6^ PBMC. **(B)** Total frequency of H3N2-specific cytokine (IFN-γ, TNF, and IL-2) production within CD4^+^ and CD8β^+^ T cells was determined by intracellular cytokine staining. Cells were restimulated *in vitro* for 18 h with H3N2 (MOI 0.1) or cultured with medium. Single, double, and triple IFN-γ-, TNF-, and IL-2-expressing CD4^+^ and CD8β^+^ T cells were analyzed by Boolean gate analysis. The corrected frequency values are shown (percentage of cytokine-producing cells minus unstimulated controls). The non-specific T-cell responses at 0 dpi were further subtracted from the T-cell responses at 14, 28, and 34 dpi. **(C)** Frequencies of single- and double-cytokine producers in CD4^+^ and CD8β^+^ T cells. The mean values ± SD **(A, C)** for each group or individual data and the mean ± SD are indicated (n = 3 per group in Exp 1 or n = 6 per group in Exp 2). Comparisons between two groups were made using the Mann–Whitney test.

T-cell responses were monitored in weekly collected PBMCs. H3N2-specific responses were assessed by IFN-γ ELISpot assay (Exp 1 and Exp 2) and by intracellular staining of IFN-γ, TNF, and IL-2 (Exp 2 only) after *in vitro* restimulation of cells with H3N2 (**Figure 3**, **Supplementary Figure 6**). IFN-γ secretion in PBMCs collected from H3N2-infected animals was detectable from 6 dpi and was overall low as previously reported (38) (<116 IFN-γ-producing cells/10^6^ cells in both Exp 1 and Exp 2 on average from 6 to 7 dpi to the end of the studies, **Figure 3A**). Intriguingly, IFN-γ secretion in PBMCs following H3N2 stimulation from PRRSV-2-infected animals was observed after 13 dpi in Exp 1 (mean of 107 IFN-γ-producing cells/10^6^ cells, **Figure 3A**), with no H3N2-specific IgG detected in the serum and BAL from this group (**Figure 2A**), suggestive of cross-reactive T cells. PRRSV-2/H3N2 co-infection induced a higher IFN-γ secretion in PBMCs compared to H3N2 single infection, although the difference was not significant (**Table 1**). Consistent with IFN-γ ELISpot assay results, the frequencies of IFN-γ, TNF, and IL-2 single, double, and triple producers within the CD4^+^ and CD8β^+^ T-cell subsets were overall low after H3N2 single infection (mean < 0.5%, **Figure 3B**). These H3N2-specific T-cell responses were the strongest at 14 dpi (mean of 0.21% CD4^+^ T cells and 0.33% CD8β^+^ T cells). While the magnitude of H3N2-specific CD4^+^ T-cell responses was similar in both single-infected and co-infected animals, that of H3N2-specific CD8β^+^ T-cell responses was higher in the co-infected group compared to H3N2-infected group (**Table 1**, **Figure 3B**).

Frequencies of mono- and polyfunctional T cells (producing at least two cytokines simultaneously) were compared after H3N2 single infection and PRRSV-2/H3N2 co-infection. Although low, H3N2-specific CD4^+^ and CD8β^+^ T cell cytokine production (alone or in combination) and kinetics were similar in PBMCs in both groups (**Figure 3C**). H3N2-specific CD4^+^ T cells were predominantly IL-2 single producers, and this response was maintained over the course of H3N2 single infection (mean of 0.10%, 0.06%, and 0.09% at 14, 28, and 34 dpi, respectively). After co-infection, CD4^+^ T cell IL-2 responses also dominated but peaked at 14 dpi (mean of 0.12%) and then declined over time. H3N2-specific CD8β^+^ T cells mainly expressed IFN-γ after the single infection, and the frequency peaked at 14 dpi (mean of 0.17%). Similarly, after co-infection, the H3N2-specific CD8β^+^ IFN-γ T-cell response dominated and peaked at 14 dpi, with a higher frequency (0.23%) in comparison to the H3N2 single infection. In addition, H3N2-specific CD8β^+^ T cell IL-2 responses were also induced in this group, reaching 0.13% 14 dpi and then gradually declining over the course of infection.

These data indicate that there was a trend toward higher H3N2-specific IFN-γ responses in blood in the PRRSV-2/H3N2 co-infected animals, although this did not reach statistical significance. No difference in the cytokine polyfunctionality of the cells was detected between the single-infected and co-infected groups.

### 3.5 H3N2-specific T-cell responses in bronchoalveolar lavage

**Figure 4:**
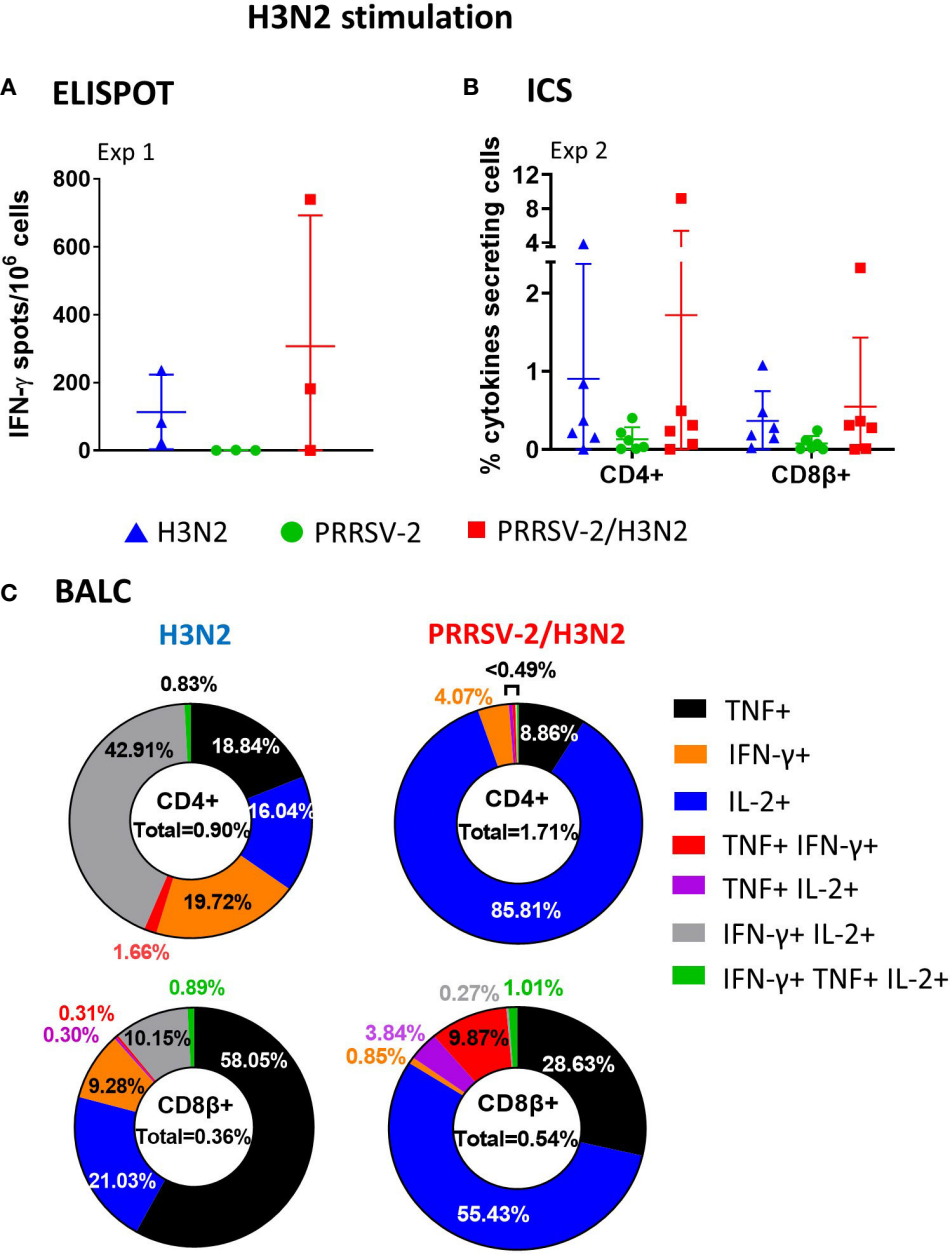
H3N2-specific polyfunctional T-cell responses in bronchoalveolar lavage fluid cells (BALCs) following PRRSV-2/H3N2 co-infection. **(A)** IFN-γ secretion by BALCs isolated at the cull (41 dpi) was determined by ELISpot assay. **(B)** Total frequency of H3N2-specific cytokines production within the CD4^+^ and CD8β^+^ T cells in BALCs collected at the end of the study (36/38 dpi) was determined by intracellular cytokine staining. Corrected frequencies of total single, double, and triple IFN-γ-, TNF-, and IL-2-producing CD4^+^ and CD8β^+^ T cells are shown. **(C)** Pie charts represent the average proportion of CD4^+^ and CD8β^+^ T cells that produced between one and three cytokines simultaneously. Individual data are indicated (n = 3 per group in Exp 1 or n = 6 per group in Exp 2). Comparisons between two groups were made using the Mann–Whitney test.

H3N2-specific T cells in BALF harvested at the end of each study (41 dpi for Exp 1; 36/38 dpi for Exp 2) were examined after *in vitro* restimulation with H3N2 (**Figure 4A**). No statistically significant differences in IFN-γ producing cells were observed between the groups, although in Exp 1, PRRSV-2/H3N2 co-infection induced slightly higher IFN-γ secretion in BALCs when compared to H3N2 single infection (mean of 307 vs. 113 IFN-γ-producing cells/10^6^ cells, respectively). In Exp 2, H3N2-specific CD4^+^ and CD8β^+^ T-cell responses in BALCs were also higher but not significantly so after PRRSV-2/H3N2 co-infection (mean of 1.71% vs. 0.90% within CD4^+^ T cells and 0.54% vs. 0.36% within CD8β^+^ T cells, **Figure 4B**).

In BALCs, the patterns of cytokine production by CD4^+^ and CD8β^+^ T cells differed between single H3N2 infection and PRRSV-2/H3N2 co-infection. Upon single infection, approximately half of H3N2-specific CD4^+^ T cells were single-cytokine producers (18.8% of TNF, 16% of IL-2, and 19.7% of IFN-γ), and the remaining 43% were double IFN-γ/IL-2 co-producers, **Figure 4C**). H3N2-specific CD8β^+^ T cells were mainly single-cytokine producers (58% of TNF, 21% of IL-2, and 9.2% of IFN-γ) followed by 10% IFN-γ/IL-2. In contrast, co-infection induced exclusively H3N2-specific single-cytokine producers CD4^+^ T cells (85% of IL-2, 8.8% of TNF, and 4% of IFN-γ, **Figure 3C**). H3N2-specific CD8^+^ T cells produced mainly one cytokine (55.4% IL-2 and 26.6% TNF), and 9.8% were double TNF/IFN-γ producers. Hence, H3N2-specific CD4^+^ and CD8^+^ T-cell responses were dominated by the production of IL-2 in the BALCs of co-infected pigs. The dominance of IL-2 may favor the survival of regulatory T cells, which may explain why co-infected animals did not show exacerbated disease (39).

In summary, there was a trend for higher IFN-γ production in BALCs from the co-infected animals, which did not reach significance. Among single cytokine-producing cells, a high proportion of IL-2-expressing CD4^+^ and CD8^+^ T cells were found after co-infection. However, both single H3N2 infection and PRRSV-2/H3N2 co-infection induced polyfunctional cytokine-producing cells T cells in BAL. CD4^+^ T cells producing both IFN-γ and IL-2 were exclusively found in the H3N2 single-infected group. CD8β^+^ T cells mainly co-expressed IFN-γ and IL-2 in the H3N2 single-infected group and TNF and IFN-γ in the co-infected group.

### 3.6 PRRSV-2-specific T-cell responses in peripheral blood

**Figure 5:**
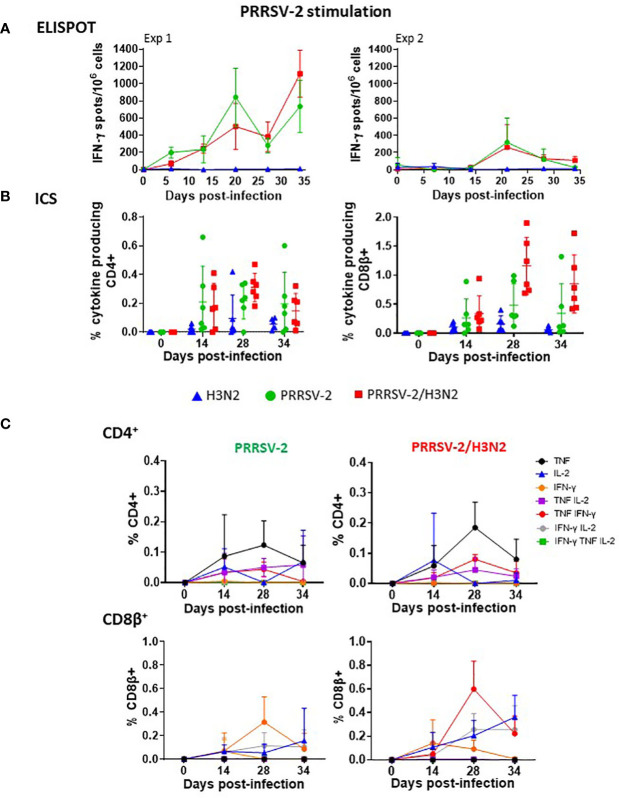
PRRSV-2-specific polyfunctional T-cell responses in peripheral blood mononuclear cell (PBMC) following PRRSV-2/H3N2 co-infection. **(A)** IFN-γ secretion by PBMCs was determined by ELISpot after stimulation with PRRSV-2 (multiplicity of infection (MOI) 3.2) or cultured with medium for 18 h. The corrected PRRSV-2-specific IFN-γ-producing cells (minus unstimulated controls) were calculated. **(B)** Total frequency of PRRSV-2-specific cytokine production within the CD4^+^ and CD8β^+^ T cells was determined by intracellular cytokine staining. Cells were restimulated *in vitro* for 18 h with PRRSV-2 (MOI 0.1) or cultured with medium. Total single, double, and triple IFN-γ-, TNF-, and IL-2-producing CD4^+^ and CD8β^+^ T cells were calculated as described in **Figure 3**. **(C)** Frequencies of single- and double-cytokine producers in CD4^+^ and CD8β^+^ T cells. The mean values + SD **(A, C)** for each group or individual data and the mean ± SD are indicated (n = 3 per group in Exp 1 or n = 6 per group in Exp 2). Comparisons between two groups were made using the Mann–Whitney test.

We next analyzed PRRSV-2-specific T-cell responses in PBMCs (**Figure 5**). PRRSV-2-specific IFN-γ secretion by PBMCs was detectable at 6 dpi increasing up to 34 dpi in Exp 1. In Exp 2, the response was undetectable up to 14 dpi, peaked at 21 dpi, and steadily decreased by the end of the study to 34 dpi (**Figure 5A**). Co-infection did not alter the PRRSV-2-specific IFN-γ response in PBMCs (**Table 1**). CD4^+^ and CD8β^+^ T-cell responses to PRRSV-2 were detectable in Exp 2 from 14 dpi onward (**Figure 5B**). Frequencies of PRRSV-2-specific CD4^+^ T-cell responses were comparable between PRRSV-2 single-infection and PRRSV-2/H3N2 co-infection groups (**Table 1**, **Figure 5B**). However, PRRSV-2-specific CD8β^+^ T-cell responses were superior in co-infected animals (*p* < 0.05, **Table 1**, **Figure 5B**).

Frequencies of mono- and polyfunctional PRRSV-2-specific T cells in PBMCs were compared between PRRSV-2 single-infection and PRRSV-2/H3N2 co-infection groups (**Figure 5C**). PRRSV-2-specific CD4^+^ T-cell TNF responses dominated and peaked at 28 dpi (mean of 0.12% and 0.18% in single-infected and co-infected groups, respectively) and declined at 34 dpi. Low proportions of CD4^+^ double-cytokine producers were mounted after PRRSV-2 single infection and PRRSV-2/H3N2 co-infection. PRRSV-2-specific CD8β^+^ T cells were mainly IFN-γ or IL-2 or double IFN-γ^/^IL-2 producers after the single infection. After co-infection, the main CD8β cytokine producers were TNF^+^IFN-γ^+^, which peaked at 28 dpi. In addition, PRRSV-2-specific CD8β^+^ T cells expressing IL-2 or IFN-γ/IL-2 gradually increased, reaching 0.25% by 34 dpi.

These results indicate that PRRSV-2/H3N2 co-infection induced an increase in IFN-γ producing CD8β^+^ T cells in the blood, dominated by double TNF/IFN-γ producers. CD4^+^ responses were comparable between the single-infected and co-infected groups and dominated by single TNF-producing cells.

### 3.7 PRRSV-2-specific T-cell responses in bronchoalveolar lavage

**Figure 6:**
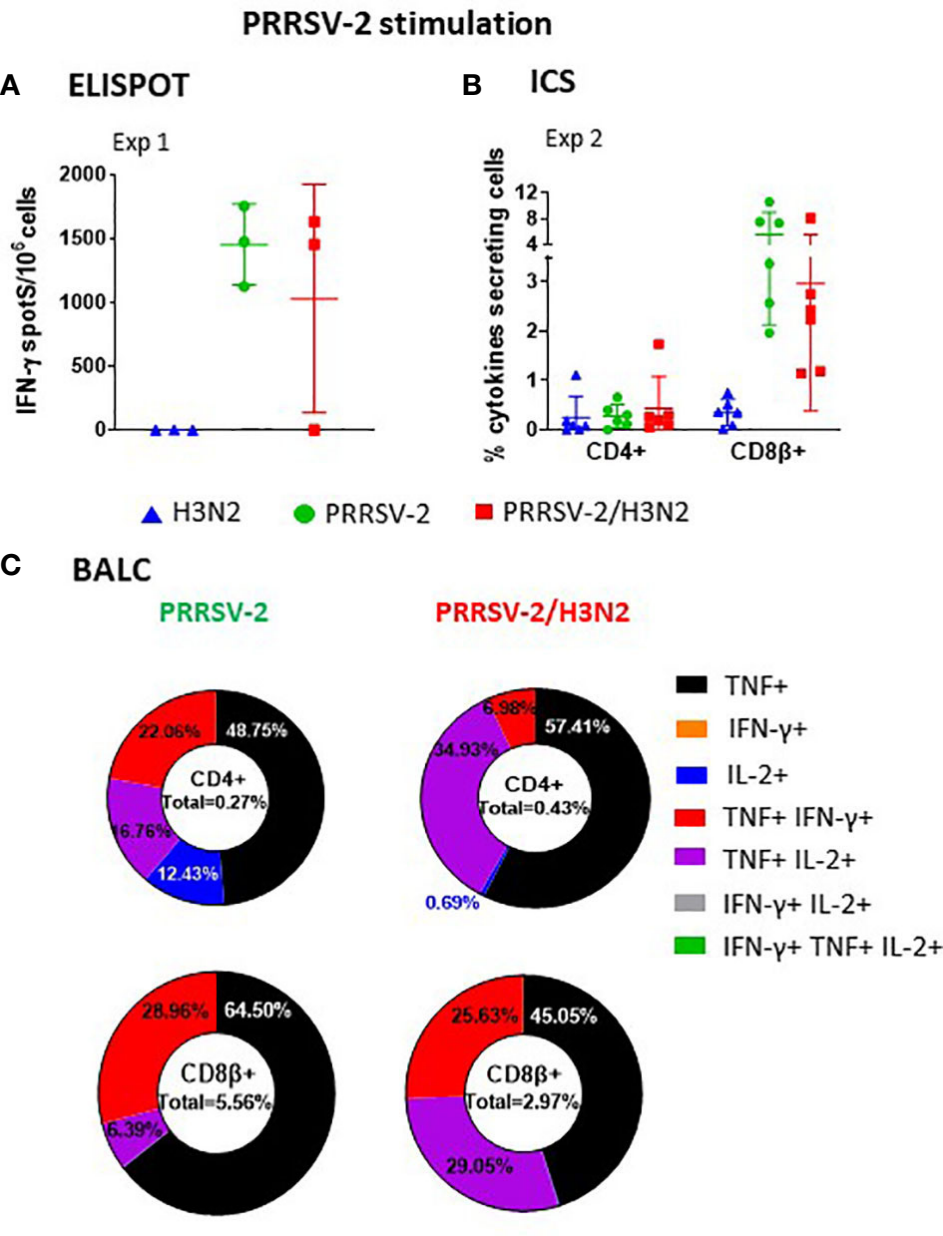
PRRSV-2-specific polyfunctional T-cell responses in bronchoalveolar lavage fluid cells (BALCs) following PRRSV-2/H3N2 co-infection. **(A)** IFN-γ secretion by BALCs isolated at the cull (41 dpi) was determined by ELISpot assay. **(B)** Total frequency of PRRSV-2-specific cytokines production within the CD4^+^ and CD8β^+^ T cells in BALCs collected at the end of study (36/38 dpi) was determined by intracellular cytokine staining as described in **Figure 3**. Corrected frequencies of total single, double, and triple IFN-γ-, TNF-, and IL-2-producing CD4^+^ and CD8β^+^ T cells are shown. **(C)** Pie charts represent the mean proportion of single, double, and triple cytokine producers in CD4^+^ and CD8β^+^ T cells. Individual data are indicated (n = 3 per group in Exp 1 or n = 6 per group in Exp 2). Comparisons between two groups were made using the Mann–Whitney test.

PRRSV-2-specific T-cell responses were assessed in BALCs at 41 (Exp 1) and 36/38 (Exp 2) dpi (**Figure 6**). IFN-γ responses as assessed by ELISpot assay were similar between the groups (**Figure 6A**). Following PRRSV-2/H3N2 co-infection, CD4^+^ T-cell responses in both BALCs were slightly enhanced (from 0.27% to 0.43% of cytokines producing CD4^+^ T cells, **Figure 6B**), although this did not reach statistical significance. High frequencies of PRRSV-2-specific CD8β^+^ T cells were identified. However, PRRSV-2/H3N2 co-infection decreased the magnitude of CD8β^+^ T-cell responses (mean of 5.56% and 2.97% between single-infected and co-infected groups, respectively).

BALCs from PRRSV-2 single-infected and PRRSV-2/H3N2 co-infected animals displayed similar patterns of polyfunctional cytokine-producing cells. Most of the PRRSV-2-specific T cells were single-cytokine producers (**Figures 6C, D**). PRRSV-2-specific TNF^+^ CD4^+^ T cells dominated (48.75% and 57.41% in PRRSV-2 single-infected and PRRSV-2/H3N2 co-infected animals, respectively, **Figure 6C**). The remaining PRRSV-2-specific CD4^+^ T cells were predominantly double TNF/IL-2 (16.76% and 34.93% in PRRSV-2 single-infected and PRRSV-2/H3N2 co-infected animals, respectively) and TNF/IFN-γ (22.06% and 6.98% in PRRSV-2 single-infected and PRRSV-2/H3N2 co-infected animals, respectively) producers (**Figure 6C**). High frequencies of PRRSV-2-specific CD8β^+^ T cells expressing TNF were observed in PRRSV-2 single-infected and PRRSV-2/H3N2 co-infection groups (64.50% vs. 45.05%, respectively), followed by double TNF/IL-2 (6.39% vs. 29.05%, respectively) and TNF/IFN-γ (28.96% vs. 25.63%, respectively) producers.

These results suggest that PRRSV-2/H3N2 co-infection did not affect significantly the local BALC response, with most of the PRRSV-2-specific CD4^+^ and CD8β^+^ T cells being single TNF producers.

## 4 Discussion

While pathogen co-infection is frequently responsible for respiratory diseases in pigs (3), most experimental studies have assessed immunopathology following a single pathogen infection. Exploration of synergistic effects or mutual interference induced toward each co-infecting agent could provide insights into the pathobiology that naturally occurs. We, therefore, assessed the consequences of swIAV and PRRSV-2 co-infection, two major contributors of PRDC, on clinical features, virus loads, and adaptive immune responses in pigs. Although many combinations of the timing of infection and virus strains are possible, we chose to simultaneously challenge pigs with contemporary field strains of swIAV H3N2 and PRRSV-2 and focused investigations on the subsequent host immune responses.

We confirmed in this study that these strains induced mild-to-moderate disease as previously observed (12), and symptoms after a concurrent PRRSV-2/H3N2 infection were not significantly different from those after the single infection. This observation is consistent with previous reports describing PRRSV-swIAV co-infection as a subclinical disease (13, 16) but also contrasts with other studies reporting a noticeable increase in fever duration and lung lesions after co-infection with these two viral species (14–16, 40). Different timings of infection, dose, virus strains, genetic background, and route of infection employed may contribute to such variable clinical outcomes, and this has been largely discussed among co-infection studies (41). Of note, increasing the H3N2 challenge dose between experiments (Exp 1 vs. Exp 2) might have affected the magnitude of specific immune responses to both co-infecting viruses but not the overall severity of the disease.

Distinct antiviral immune responses are mounted after infection with swIAV or PRRSV-2. During the early phase of swIAV infection, pro-inflammatory cytokines (IFN-α, TNF, IL-12, and IL-6) are secreted in the lung along with infiltration of immune cells including neutrophils, dendritic cells (DCs), NK cells, and macrophages (42–46). This acute inflammatory response, if not tightly regulated, is associated with lung injury and respiratory distress (47, 48). Likewise, PRRSV-2 induces a local secretion of proinflammatory cytokines (IL-1β, IL-6, IL-8, IL-12, IFN-α, and TNF) and infiltration of DCs and macrophages in the lung (49). However, PRRSV-2 has the ability to subvert the host immune system by activating regulatory T cells secreting immunosuppressive cytokines (IL-10 and TGF-β) (50, 51), decreasing NK cell degranulation capacity (52) and antagonizing type I IFN signaling pathways (53–55). We questioned whether PRRSV-2-mediated immune modulation would favor swIAV H3N2 replication and subsequently enhances the severity of the disease. However, we showed that swIAV H3N2 replication was reduced in the lungs during co-infection in agreement with our previous co-infection study (12). Our observation may be attributable to a high, regulated, inflammatory response at the site of viral replication upon co-infection, which led to an effective local antiviral response impairing viral replication. Indeed, a synergistic increase of TLR3, RIG-I, and IFN-β transcripts was demonstrated after concomitant *in vitro* infection of lung slices with swIAV H1N1 and PRRSV-2, although the level of replication of either virus was not reduced compared to single-infected conditions (56). Another possible molecular mechanism may involve an alteration of AMP-activated protein kinase (AMPK) signaling by PRRSV, ultimately affecting swIAV replication (41). AMPK regulates autophagy (57), which promotes IAV replication (58). PRRSV-2 RNA loads were also reduced upon co-infection despite the variability between studies (12). Further investigations are needed to fully elucidate molecular mechanisms involved in this complex interplay between co-infecting viruses and identify what component can act toward detrimental clinical outcomes.

We questioned whether PRRSV/swIAV co-infection influences the immune responses against each other, which potentially leads to an altered outcome of infection. Probably due to a relatively low number of samples per group and high variability in gene responses among groups, there were no significant differences in gene expression observed between the groups at 5 dpi. This is suggestive of similar innate immune gene expression between groups, an inadequate time, and a type of sampling (whole blood instead of the lung) to capture relevant changes in gene expression (59). Serum H3N2-specific IgG and neutralizing antibody titers were higher in the co-infected group, indicating that PRRSV-2/H3N2 co-infection did not impair the establishment and magnitude of antibody responses toward H3N2, in agreement with previous studies (11, 14). Polyfunctional CD8^+^ T cells are associated with effective viral clearance (60). We showed that PRRSV-2/H3N2 co-infected animals displayed more diverse polyfunctional H3N2-specific CD8β^+^ T-cell responses in BALCs, compared to the H3N2 single-infected group. We found H3N2-specific CD8β^+^ T cells co-expressing TNF and IFN-γ in BALCs from the co-infected but not the single-infected group. It is possible that these are cytotoxic CD8β^+^ T cells secreting TNF and IFN-γ that might be involved in controlling H3N2 replication in the lung. Such CD8^+^ T cells are highly efficient to elicit degranulation upon antigen stimulation *in vitro* (60). Similarly, a higher proportion of H3N2-specific polyfunctional CD8β^+^ T cells were found in PBMCs from co-infected pigs. PRRSV-2-specific CD8β^+^ T cells co-expressing TNF and IL-2 were also detected in BALCs of the co-infected groups, although these were not associated with a decrease of PRRSV-2 RNA loads in serum or BALF.

Previously, we have shown that simultaneous PRRSV/swIAV co-infection abrogated the clinical protection induced by live attenuated PRRSV vaccine (12). In the present study, pigs concurrently co-infected with swIAV H3N2 and PRRSV-2 mounted comparable and, in some cases, superior immune responses to the co-infecting viruses, compared to those elicited by a single virus infection. These observations raise questions as to which co-infection factors may be responsible for the aggravated respiratory disease since immune responses do not appear to be unduly perturbed at least by PRRSV-2/H3N2 infection.

## Data availability statement

The datasets presented in this study can be found in online repositories. The names of the repository/repositories and accession number(s) can be found below: PRJNA940926 (SRA).

## Ethics statement

The animal study was reviewed and approved by Animal Welfare and Ethical Review Board (Pirbright institute).

## Author contributions

ET, SG, NW, and SK acquired funding for the project. ET, SG, and TC contributed to the conception, design, and coordination of the study. TC, EM, KR, EV, AM, TM, and GB performed the experiments. TC acquired, analyzed, and interpreted the data. EM, KR, EV, BP, AM, VM, TM, EB, BVC, BC, ME, SG, and ET contributed to the post-mortem sampling. NP and GF conducted RNA-sequencing. AS performed bioinformatics analysis. AN conducted the pathological analysis. TC wrote the first draft of the manuscript. ET, SG, AS, and GF edited and revised the manuscript. All authors contributed to the article and approved the submitted version.
