# Supplementary material for: Simultaneous co-infection with swine influenza A and porcine reproductive and respiratory syndrome viruses potentiates adaptive immune responses
Source: Front Immunol. 2023 May 23;14:1192604. doi: 10.3389/fimmu.2023.1192604 (PMC10242126; doi:10.3389/fimmu.2023.1192604)
Supplement: Supplementary file 1 [file DataSheet_1.pdf]

Supplementary Material

Simultaneous co-infection with swine influenza A and porcine reproductive and respiratory syndrome viruses potentiates adaptive immune responses

Tiphany Chrun[1], Emmanuel A. Maze[1], Kelly J. Roper[1], Eleni Vatzia[1], Basudev Paudyal[1], Adam McNee[1], Veronica Martini[1], Tanuja Manjegowda[1], Graham Freimanis[1], Adrian Silesian[1], Noemi Polo[1], Becky Clark[1], Emily Besell[1], Georges Booth[1], Brigid Veronica Carr[1], Matthew Edmans[1], Alejandro Nunez[2], Surapong Koonpaew[3], Nanchaya Wanasen[3], Simon P. Graham[1†]*, Elma Tchilian[1†]*

*** Correspondence:**

Simon Graham
simon.graham@pirbright.ac.uk

Elma Tchilian
elma.tchilian@pirbright.ac.uk

Supplementary Table 1. Scoring index of the clinical signs

	Signs	Score
**Temperature**	<39	0
	39.0 < to < 39.5	1
	39.5 ≤ to < 40	2
	40.0 to 40.5	3
	40.6 to 41	4
	>41	5
**Inappetence**	Reduced eating	1
	Only picking at food	4
	Not eating	6
**Recumbancy**	Lethargic	1
	Get up only when touched	2
	Slow to get up when touched	4
	Remain recumbent when touched	6
**Skin Haemorrhage**	Blue/purple-discoloured on ears and/or body	1
	Haemorrhagic areas on ears and body	2
	Generalised haemorrhage all over body	3
**Respiratory changes**	Laboured breathing and/or frequent coughing	2
	Severe – breathing through open mouth	6
**Nasal discharge**	Present and clear	1
	Present and discoloured	2
**Eyes/conjunctiva**	Ocular discharge (gummed up eyes)	1
	Swelling of eyelids	1
**Body condition**	Fair	1
	Poor (ribs/backbone showing)	2

Supplementary Figure 1

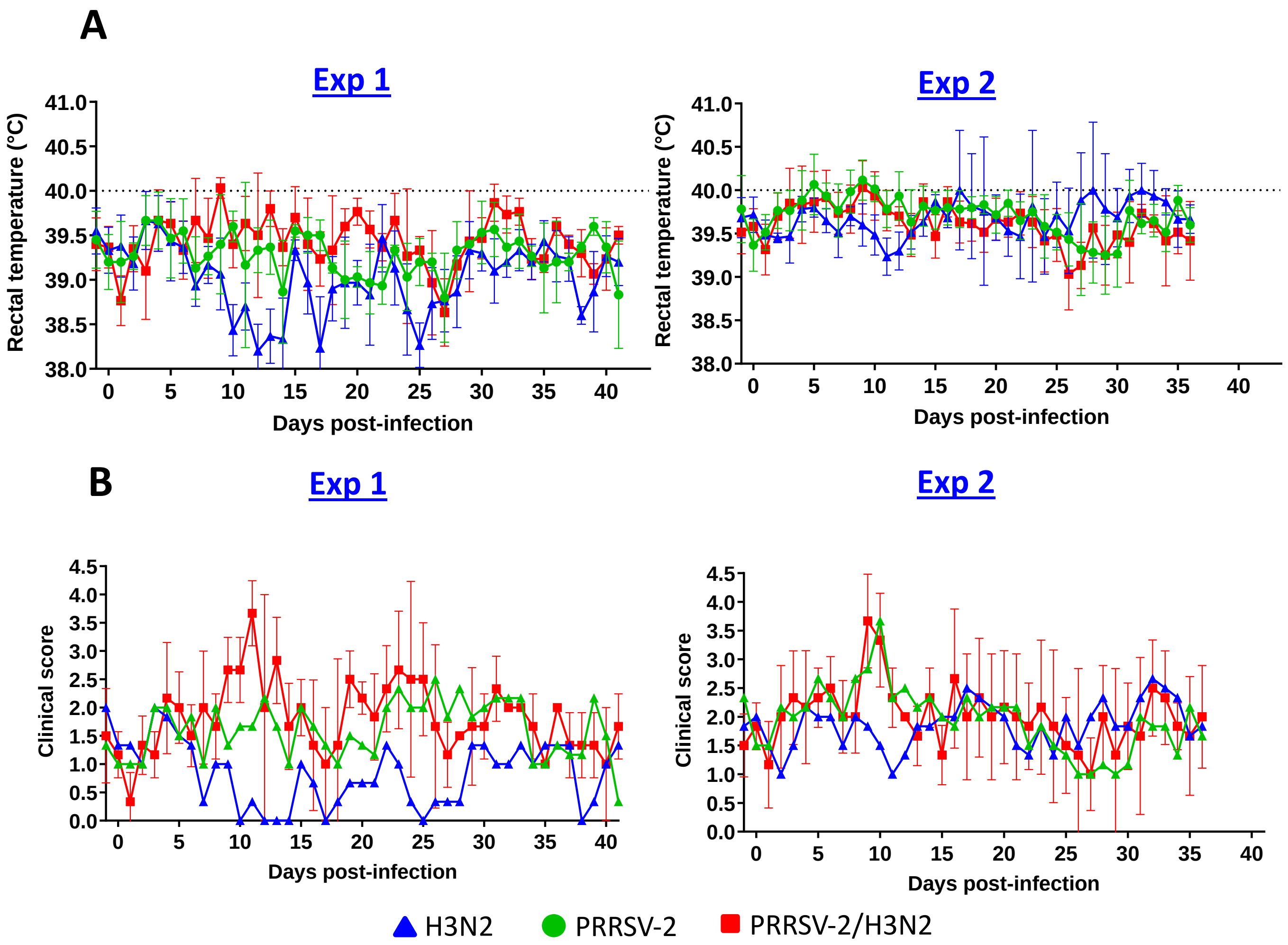

Supplementary Figure 1. Rectal temperature and clinical score. Mean (±SD) of the rectal temperature (°C) **(A)** and the clinical score **(B)** following single and co-infection with H3N2 and PRRSV-2 for each experiments (Exp). The dashed line in graphs **(A)** indicates the temperature baseline (40°C).

Supplementary Figure 2

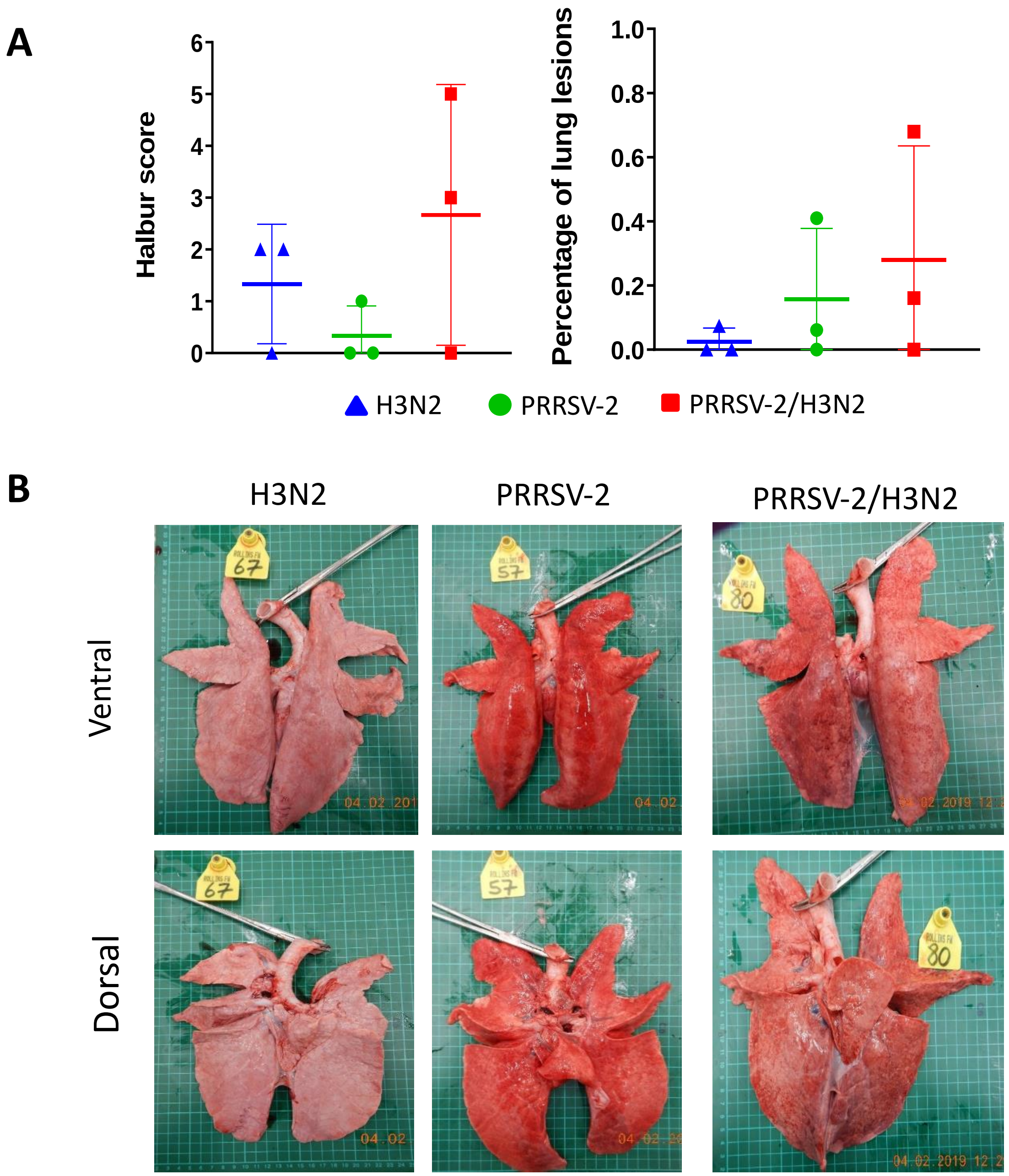

Supplementary Figure 2. Gross pulmonary lesions. Lungs of pigs challenged with H3N2, PRRSV-2 or simultaneously with both viruses by intranasal inoculation were dissected out at 5 dpi (n=3 per group). Digital images were taken for semi-quantitative (Halbur score) and quantitative (percentage of gross lung lesions) assessment. The score of each animal and the mean ± SD are displayed in the top panel **(A)**, and representative images of the lungs (ventral and dorsal view) from each group are shown in the lower panel **(B)**.

Supplementary Figure 3

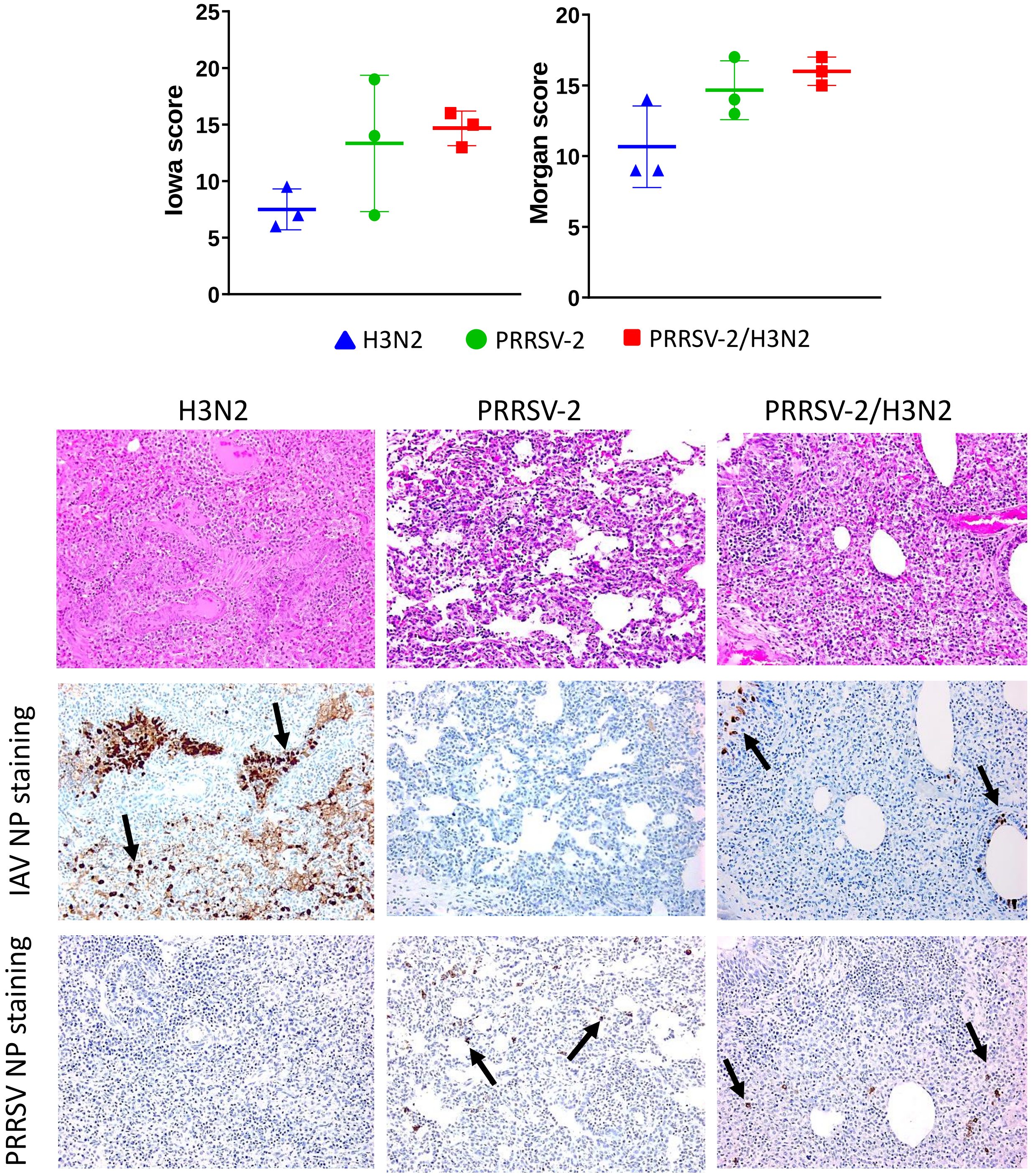

Supplementary Figure 3. Histopathology of lungs. Sections of cranial, cardiac and diaphragmatic lung lobes collected at 5 dpc were stained with H&E, mAb against IAV nucleoprotein or mAb against. Microscopic lesions scored as per Iowa and Morgan score. **(A)** The score of each animal and the mean ± SD are displayed (n=3 per group). **(B)** Representative images of histopathology **(B)** and immunohistochemical images **(C)** of the lung samples from each group are shown. Presence of IAV NP- or PRRSV-NP positive cells are indicated by the black arrows.

Supplementary Figure 4

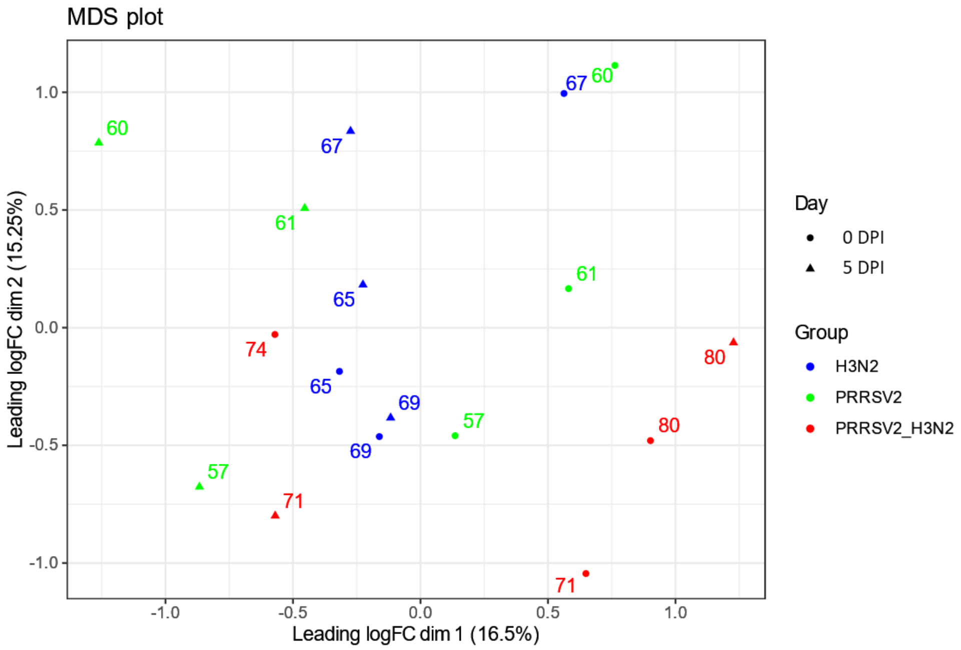

Supplementary Figure 4. Multidimensional scaling plot. LogFC method is used to calculate the distance between samples based on log2 fold changes of the top 500 genes. Only two first dimensions with corresponding percentage of variance explained are shown.

Supplementary Figure 5

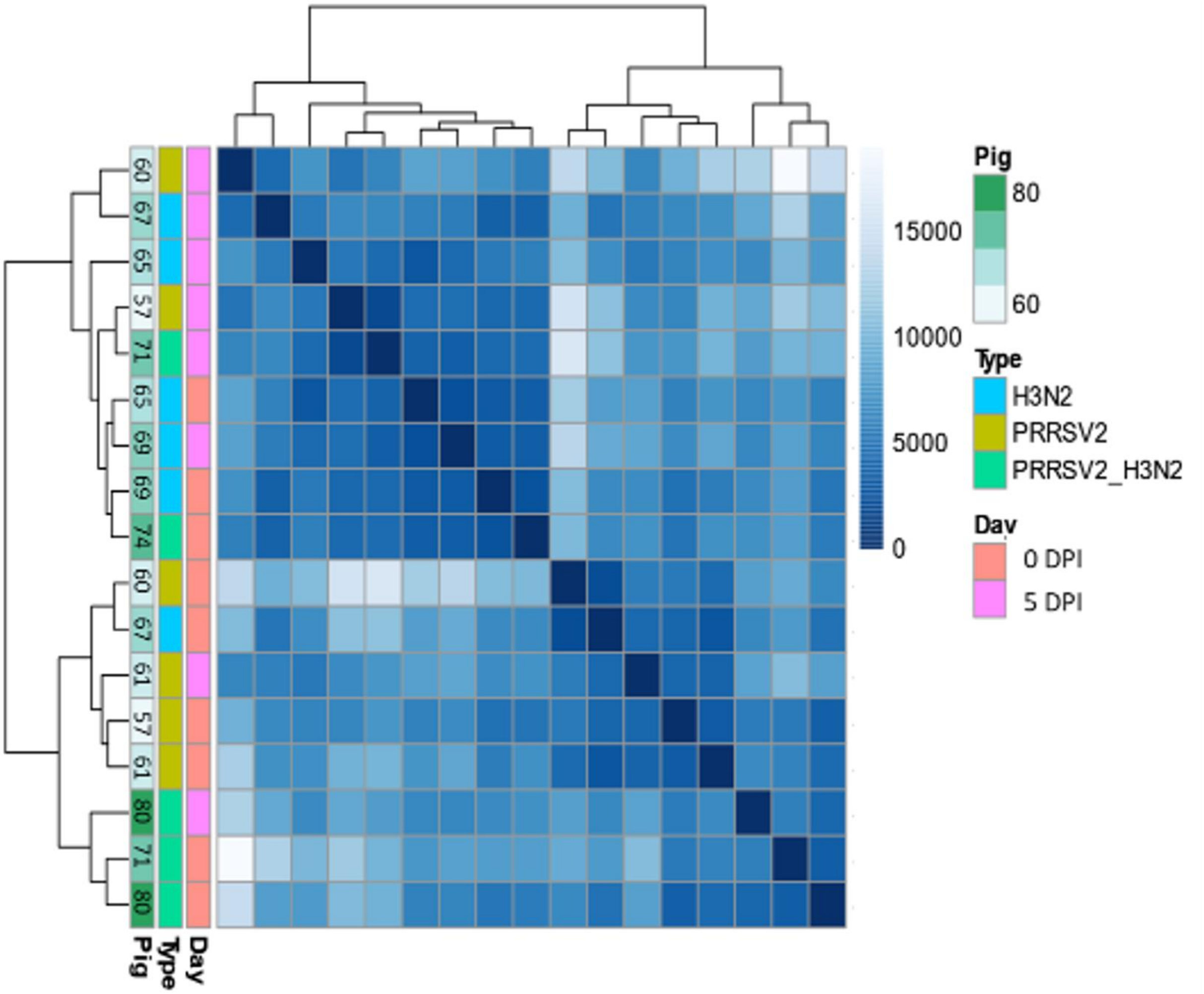

Supplementary Figure 5. A hierarchical clustering of sample to sample distance calculated based on the Poisson distance of normalized counts.

Supplementary Figure 6

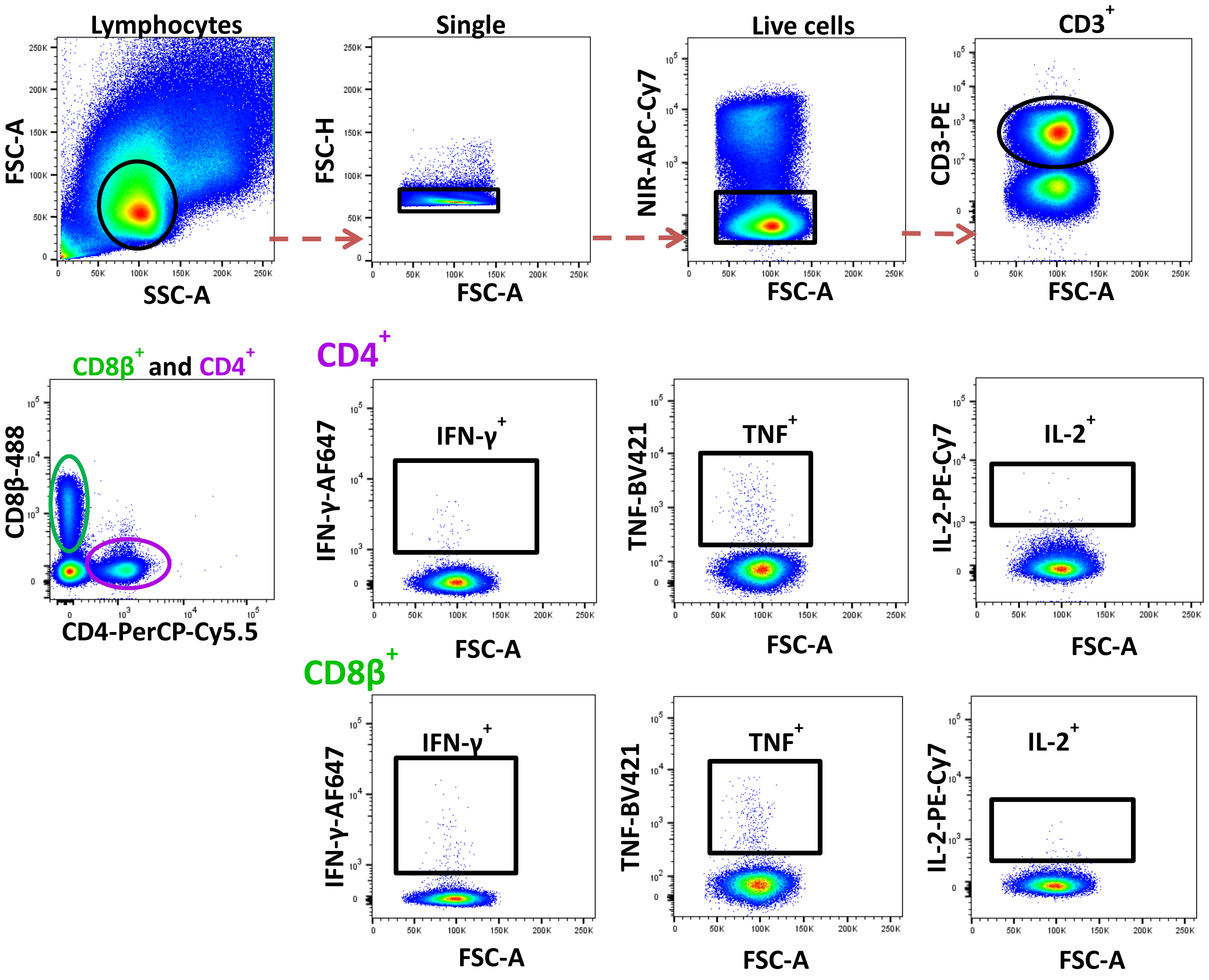

Supplementary Figure 6. Intracellular cytokine staining gating strategy. Gating was done on lymphocytes, single cells, negative cells for live/dead viability dye, $CD3^+$ T cells and $CD4^+$ or $CD8\beta^+$ T cells. Expression of IFN-γ, TNF and IL-2 was further analysed for each T cell subset. Representative plots are shown.
